# Acute blockade of endogenous melatonin by Luzindole, with or without peripheral LPS injection, induces jejunal inflammation and morphological alterations in Swiss mice

**DOI:** 10.1590/1414-431X2021e11215

**Published:** 2021-08-20

**Authors:** R.S. Matos, R.B. Oriá, P.F.C. Bruin, D.V. Pinto, A.F.S.C. Viana, F.A. Santos, A.S.G. Duarte, V.M.S. Bruin

**Affiliations:** 1 Laboratório de Sono e Ritmos Biológicos, Faculdade de Medicina, Universidade Federal do Ceará, Fortaleza, CE, Brasil; 2 Laboratório de Produtos Naturais, Faculdade de Medicina, Universidade Federal do Ceará, Fortaleza, CE, Brasil; 3 Laboratório da Biologia da Cicatrização, Ontogenia e Nutrição de Tecidos, Faculdade de Medicina, Universidade Federal do Ceará, Fortaleza, CE, Brasil; 4 Departamento de Morfologia, Faculdade de Medicina, Universidade Federal do Ceará, Fortaleza, CE, Brasil

**Keywords:** Luzindole, Melatonin, LPS, Inflammation, Oxidative stress, Intestine

## Abstract

This study investigated the acute blockade of endogenous melatonin (MLT) using Luzindole with or without systemic lipopolysaccharide (LPS) challenge and evaluated changes in inflammatory and oxidative stress markers in the mouse jejunum. Luzindole is an MT1/MT2 MLT receptor antagonist. Both receptors occur in the small intestine. Swiss mice were treated with either saline (0.35 mg/kg, *ip*), Luzindole (0.35 mg/kg, *ip*), LPS (1.25 mg/kg, *ip*), or Luzindole+LPS (0.35 and 1.25 mg/kg, *ip*, respectively). Jejunum samples were evaluated regarding intestinal morphometry, histopathological crypt scoring, and PAS-positive villus goblet cell counting. Inflammatory Iba-1, interleukin (IL)-1β, tumor necrosis factor (TNF)-α, nuclear factor (NF)-kB, myeloperoxidase (MPO), and oxidative stress (NP-SHs, catalase, MDA, nitrate/nitrite) markers were assessed. Mice treated with Luzindole, LPS, and Luzindole+LPS showed villus height shortening. Crypt damage was worse in the LPS group. Luzindole, LPS, and Luzindole+LPS reduced the PAS-goblet cell labeling and increased Iba-1-immunolabelled cells compared to the saline group. Immunoblotting for IL-1β, TNF-α, and NF-kB was greater in the Luzindole group. The LPS-challenged group showed higher MPO activity than the saline and Luzindole groups. Catalase was reduced in the Luzindole and Luzindole+LPS groups compared to saline. The Luzindole group showed an increase in NP-SHs, an effect related to compensatory GSH activity. The acute blockade of endogenous MLT with Luzindole induced early changes in inflammatory markers with altered intestinal morphology. The other non-detectable deleterious effects of Luzindole may be balanced by the unopposed direct action of MLT in immune cells bypassing the MT1/MT2 receptors.

## Introduction

Melatonin (5 methoxy-N-acetyltryptamine, MLT) is an ancestral indoleamine, widely present in mammals and other living beings as plants. Its widespread presence suggests an important role in tissue homeostasis. MLT was first identified in the brain, being produced primarily by the pineal gland, with its secretion being regulated by the medial geniculate body (1).

However, MLT is also produced by enterochromaffin cells of the digestive mucosa, and previous evidence has indicated a higher concentration of MLT in the gastrointestinal tract (about 400 times greater) than in the pineal gland. Indeed, that tissue can be one of the main sites responsible for maintaining the plasma levels of MLT. In the gastrointestinal tract, MLT plays an important role in regulation of motility and anti-inflammatory responses (2).

The mechanisms of action of MLT are largely mediated by its MT receptors (3). MLT and its receptors MT1/MT2 are highly expressed in the small intestine. MT1 is mainly found in the cytoplasm of epithelial tuft cells, goblet cells, and enterocytes. The most prominent MT2 expression is found in lining epithelium, particularly in the enterocyte nucleus and cytoplasm throughout the gastrointestinal tract, with the strongest expression seen in the large intestine. MT1/MT2 are also found in the submucosal tissue, myenteric plexus, and in blood vessels (2,4).

Cumulative evidence indicates that MLT actions are not only mediated by the more widespread membrane receptors MT1/MT2. MLT may also bypass MT1/MT2, by having a direct action due to its lipophilic properties, throughout the cell membrane to the cell nucleus or cytoplasm or by binding to MT3, another MT receptor (5,6).

MLT is recognized as a potential scavenger with antioxidant and anti-inflammatory activities (7,8). The importance of exogenous MLT in the intestine is further supported by the evidence that the light-dark cycle influences inflammation and antioxidant activity (4). However, little is known about endogenous intestinal MLT functions and regulation.

This study addressed whether early blocking of endogenous MLT by Luzindole, an MT1/MT2 inhibitor, compromises intestinal homeostasis, thereby, evaluating the MT1/MT2-dependent constitutive role of MLT on the small intestine. The Luzindole-blocking effects were compared with the acute actions of systemic lipopolysaccharide (LPS) challenge, alone or in combination.

## Material and Methods

### Chemicals

LPS (L2630, *Escherichia coli* 0111:B4), Luzindole (L2407), and dimethyl sulfoxide (DMSO) were purchased from Sigma-Aldrich (USA). LPS was diluted in sterile phosphate-buffered saline (PBS). Luzindole was diluted in DMSO.

### Animals

Experiments in this study were all approved by the Ethics Committee for Animal Care from the Universidade Federal do Ceará (UFC) (protocol number 117/2016). Thirty-two male Swiss mice (25-30 g) were obtained from the Department of Surgery vivarium, UFC (Brazil). The animals were maintained under standardized temperature and light conditions (12-h light/dark cycle; temperature, 24±2°C). Experimental mice had access to tap water and food *ad libitum*.

### Experimental protocol

The animals were randomly assigned to four experimental groups: mice receiving either saline solution (0.35 mg/kg, *ip*, n=8), Luzindole (0.35 mg/kg, *ip*, n=8), LPS (1.25 mg/kg, *ip*, n=8), or Luzindole+LPS (0.35 mg/kg, *ip* and 1.25 mg/kg, *ip*, respectively, n=8). All studied animals were euthanized by an overdose of xylazine and ketamine solution, 1.5 h after receiving saline or treatment. After laparotomy, the jejunum was removed. One segment was harvested and immersed in formaldehyde for histological analyses and another segment was snap-frozen in liquid nitrogen and soon after stored in a -80°C freezer for intestinal oxidative stress and myeloperoxidase (MPO) analyses.

The dose of Luzindole (0.35 mg/kg, *ip*) and the administration time for studying its acute effects on the small intestine was based on Sommansson et al. (9). The authors observed that 0.35 mg/kg was sufficient to block MT1/MT2 receptors in the mouse gut. The dose of LPS (1.25 mg/kg), as well as acute treatment time and euthanasia (1.5 h), were based on the experimental model of apoptosis and diarrhea from Williams et al. (10). Williams and colleagues confirmed that the time-lapse of 1.5 h following LPS injection (1.25 mg/kg, *ip*) was sufficient to cause villus blunting with high rates of cell apoptosis and diarrhea, therefore indicating detectable acute mucosal tissue injury.

### Morphometric analysis

In order to assess the effect of acute endogenous MLT blockage, we analyzed intestinal morphometric parameters and goblet cell activity as markers of intestinal mucosa integrity.

#### Villus height

After euthanasia, jejunum samples were removed and fixed in 10% buffered formalin for 24 h and then stored in 70% alcohol. Soon after, samples were dehydrated in alcohol and fixed in paraffin. The sections were stained with hematoxylin-eosin (H&E) for villus height measurement, which is a marker of intestinal tissue absorptive surface. Villus height was measured from the baseline to the top in clear transversal sections. Ten villi per animal were analyzed using an optical microscope (Olympus CX3, Japan) and an image acquisition system (Q-Color 3, Olympus). Measurements were taken from captured digitalized images using ImageJ 1.4^®^ software (NIH, USA) after proper calibration. All analyses were done blindly by an experienced histologist.

### Crypt necrotic scores

Crypt necrotic scores were determined by light microscopy (Olympus CX31) in H&E slides at both low and high magnification by an experienced histologist, who was blinded to the experimental treatments. Table 1 lists the criteria used for the evaluation and scoring of intestinal crypts (11). A lower score indicates normal crypt architecture with secretory cells showing conspicuous cytoplasmic granules.

**Table 1:** Criteria used for the evaluation and scoring of intestinal crypts.

	Histopathological characteristics
Score 0	>90% of crypts showing normal cytoarchitecture and size without enlargement and hyperplasia. Well-defined gland base. Intact Paneth and goblet cells with conspicuous cytoplasmatic granules.
Score 1	>70% exhibiting crypt hyperplasia and enlargement. Normal cytoarchitecture without necrotic change. Intact Paneth and goblet cells with conspicuous cytoplasmatic granules.
Score 2	Similar to score 1, however 30% of the normal crypts showing cytoarchitecture changes and necrotic lesion at its base. Infiltration of inflammatory cells, with the possible presence of Paneth and goblet cells but without conspicuous cytoplasmatic granules.
Score 3	Between 50 and 70% crypts exhibiting necrotic changes at the base of the crypt with infiltration of inflammatory cells. The remaining crypts show hyperplasia and enlargement. Absence of Paneth and goblet cells.
Score 4	>70% crypts exhibiting necrotic changes at the base with infiltration of inflammatory cells. Absence of Paneth and goblet cells.

Source: reference 11 (doi: 10.1590/1414-431X20144360).

### Goblet cell counting

In order to detect the mucus present in goblet cells of mouse villus, specimens were subjected to 4-μm-thick cross-sections and mounted onto glass slides, dewaxed, rehydrated, and oxidized with 0.5% periodic acid. Jejunum sections were stained with Schiff's reagent for 20 min, and subsequently, the same slides were back-stained with hematoxylin for 20 min. Images were obtained with the aid of an optical microscope (Olympus CX3) and an image acquisition system (Q-Color 3, Olympus). The number of goblet cells was counted in eight to ten villi per animal. All the analyses were done blindly by an experienced histologist.

### Evaluation of inflammatory markers

#### Immunohistochemistry for Iba-1, IL-1β, TNF-α, and NF-kB.

Jejunal samples were cross-sectioned at 4-µm thickness and mounted onto glass slides coated with organosilane-based adhesive (Sigma Aldrich). The sections were immunostained using Iba-1, interleukin (IL)-1β, tumor necrosis factor (TNF)-α, and nuclear factor (NF)-kB antibodies (Santa Cruz Biotechnology, Interprise, Brazil), by the streptavidin-biotin method (12). Briefly, the histological sections were dewaxed and rehydrated in different grades of xylol and alcohol. Antigenic retrieval was performed in a 6.0 pH in a pressure cooker, for 30 s at 126°C. Thereafter, endogenous peroxidase blockade with 3% hydrogen peroxide was performed for 10 min. Sections were then incubated with Iba-1, IL-1β, TNF-α, and NF-kB antibodies for 1 h at a dilution of 1:300 in PBS with bovine serum albumin (PBS-BSA) and then washed with PBS solution. The sections were then incubated with the secondary LSAB antibody kit (DAKO^®^, USA) for 10 min at room temperature. Then, incubation was performed in a chromogenic solution with 3,3'-diaminobenzidine (DAB) for 5 min in a dark room. The slices were then rinsed with distilled water. The immunohistochemical staining of the antigens was confirmed in cells that exhibited brownish staining in their cytoplasm, independent of the intensity of the immunostaining. Immunohistochemistry images were captured by an optical microscope (Olympus CX3) and the image acquisition system (Q-Color 3, Olympus). To quantify an immunolabeled area, ImageJ 1.4^®^ software (NIH) was used. The measurement was calculated by the ratio of the total area in relation to the immunostained fraction area (%). Four random high-magnification fields per animal were quantified.

#### Determination of intestinal myeloperoxidase (MPO)

The MPO activity was measured in the jejunum samples using a colorimetric assay as described by Bradley et al. (13). The results are reported as U/mg of tissue.

### Evaluation of oxidative stress markers

#### Determination of non-protein sulfhydryl (NP-SH) levels

The jejunum NP-SHs (reduced glutathione, GSH) were determined in tissue homogenates by Ellman's reaction using 5050-dithio-bis-2-nitrobenzoic acid (14). The results of NP-SHs are reported as µmol/mg of tissue.

#### Measurement of the catalase (CAT) activity

CAT activity was assayed in jejunum homogenates according to a modified spectrophotometric method (15). CAT activity was defined as the amount of enzyme required to decompose 1 nmol of H_2_O_2_ per minute at 25°C and pH 7, and the results are reported as mmol/min per 100 mg of tissue.

#### Determination of malondialdehyde (MDA) levels

The concentration of jejunum lipid peroxidation was determined by assessing MDA in tissue homogenates using the thiobarbituric acid test (16). The results are reported as nmol/mg of tissue.

#### Determination of nitrite/nitrate levels

Tissue levels of nitric oxide were measured as total nitrite/nitrate using Griess reagent assay (17). The results are reported as µmol/mg of protein.

### Statistical analysis

Results are reported as means±SE. Analysis of variance (ANOVA) followed by Bonferroni's test was applied to compare multiple groups. The Kruskal-Wallis test with Dunn's *post hoc* test was used to determine the significance of differences in crypt necrosis scores. P<0.05 was considered significant. All statistical analyses were carried out with GraphPad Prism version 6 (USA).

## Results

### Morphometric analyses

#### Villus height

**Figure 1:**
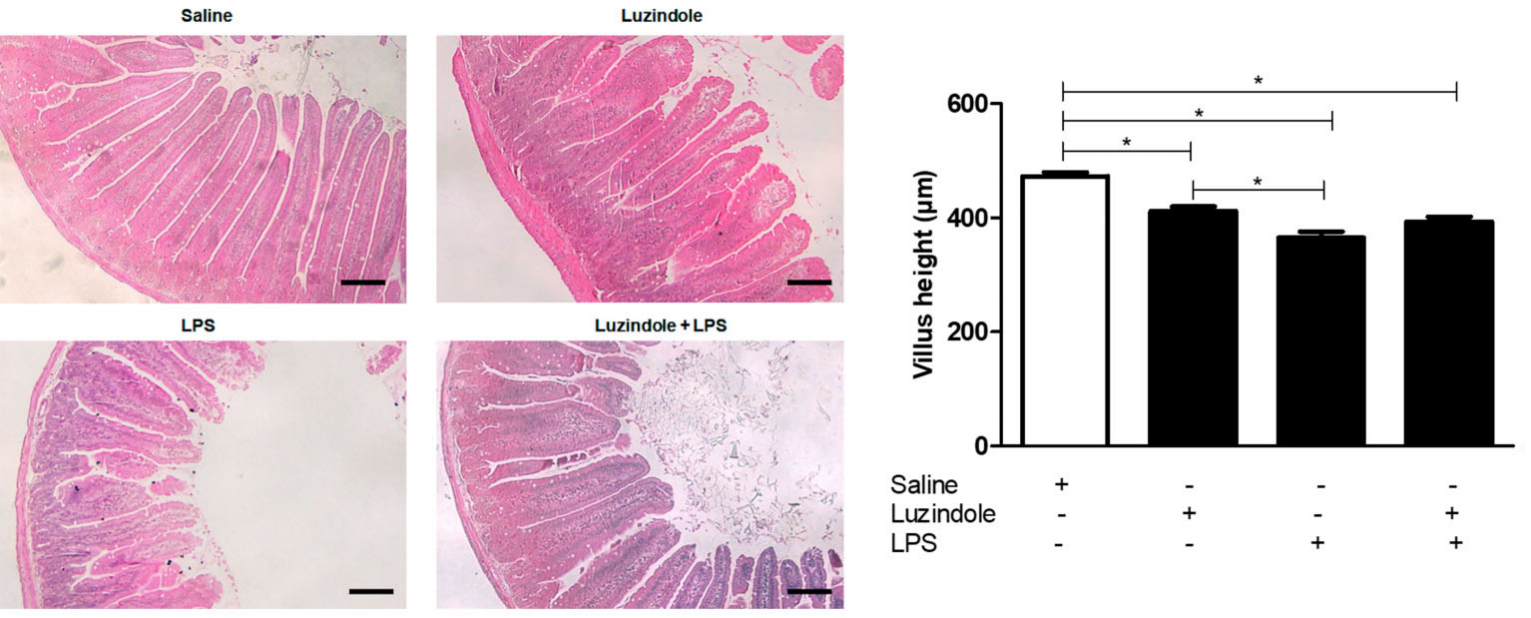
Representative histology of jejunal villus from the experimental groups treated with saline, Luzindole, lipopolysaccharide (LPS), and Luzindole+LPS. Scale bar=100 µm. Graph showing the villus height of each group. Data are reported as means±SE. *P<0.05 (ANOVA followed by Bonferroni's test).

Mice treated with Luzindole, LPS, and Luzindole+LPS showed a significant reduction of villus height compared to the saline group (P<0.05). In addition, the LPS-treated group showed blunted villi compared to the Luzindole group (P<0.05). Noteworthy, there was no difference between LPS and Luzindole+LPS groups (Figure 1).

### Crypt histopathological scores

Crypt scores from the unchallenged saline group were graded zero. The LPS group had higher scores compared to the saline, Luzindole, and Luzindole+LPS groups (P<0.05). However, no difference among the saline, Luzindole, and Luzindole+LPS groups was identified (P>0.05) (Table 2).

**Table 2:** Crypt histopathological scores of the experimental mice after treatment with saline, Luzindole, lipopolysaccharide (LPS), and Luzindole+LPS.

	Saline	Luzindole	LPS	Luzindole+LPS
Score	0 (0-0)	0 (0-1)	3 (2-4)^a^	0.5 (0-1)

Data are reported as mean and the variation (0-4). ^a^P<0.05 compared with saline, Luzindole, and Luzindole+LPS (Kruskal-Wallis test).

### Goblet cell counting

**Figure 2:**
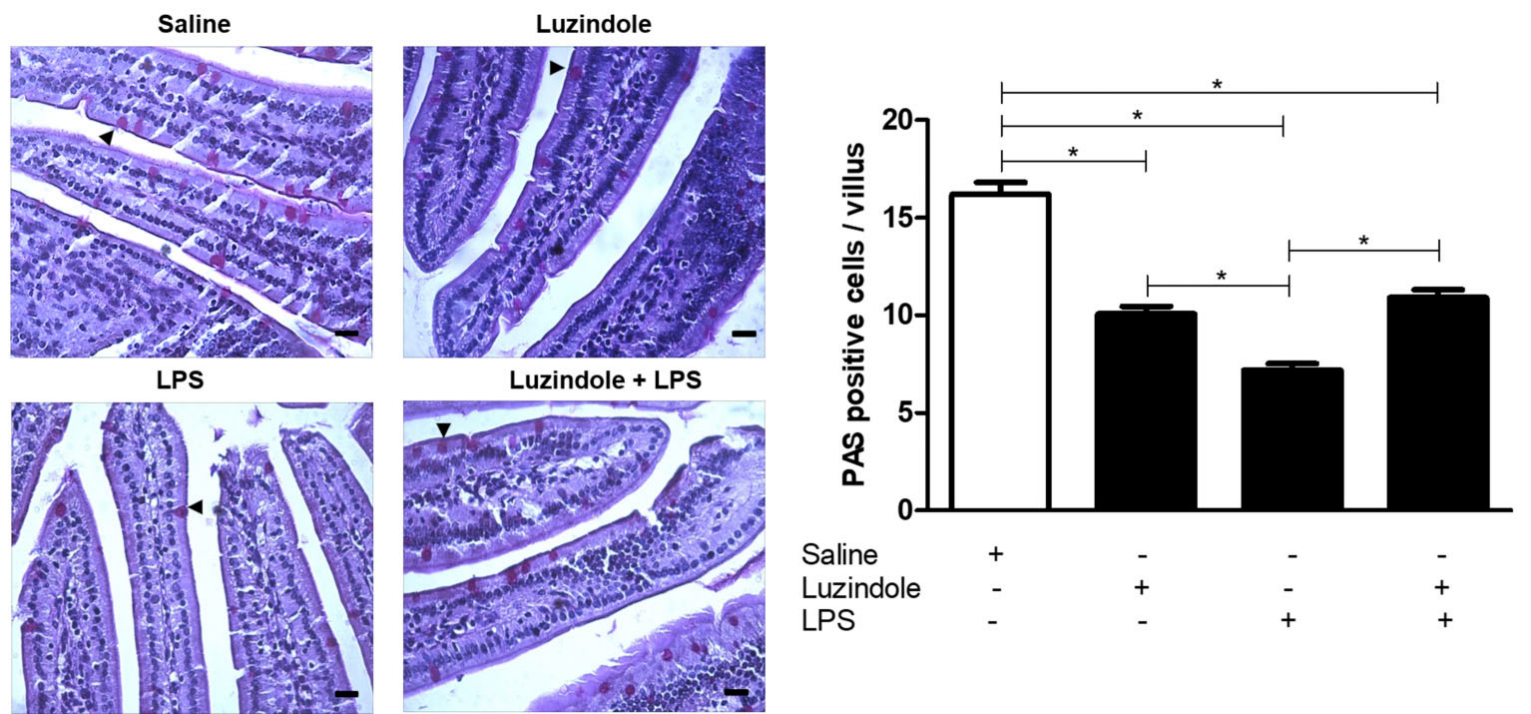
Representative histology of PAS staining in the experimental mouse jejunum after treatment with saline, Luzindole, lipopolysaccharide (LPS), and Luzindole+LPS. Arrowheads indicate goblet cells. Scale bar=20 µm. Graph showing the PAS-positive cells of each group. Data are reported as means±SE. *P<0.05 (ANOVA followed by Bonferroni's test).

The Luzindole, LPS, and Luzindole+LPS groups showed lower PAS-positive goblet cell counts compared to the saline group. Differences were also observed between the Luzindole and LPS groups, as well as LPS and Luzindole+LPS (P<0.05). No statistical difference was found between the Luzindole and Luzindole+LPS groups (P>0.05) (Figure 2).

### Inflammatory markers

#### Iba-1 immunolabeling

**Figure 3:**
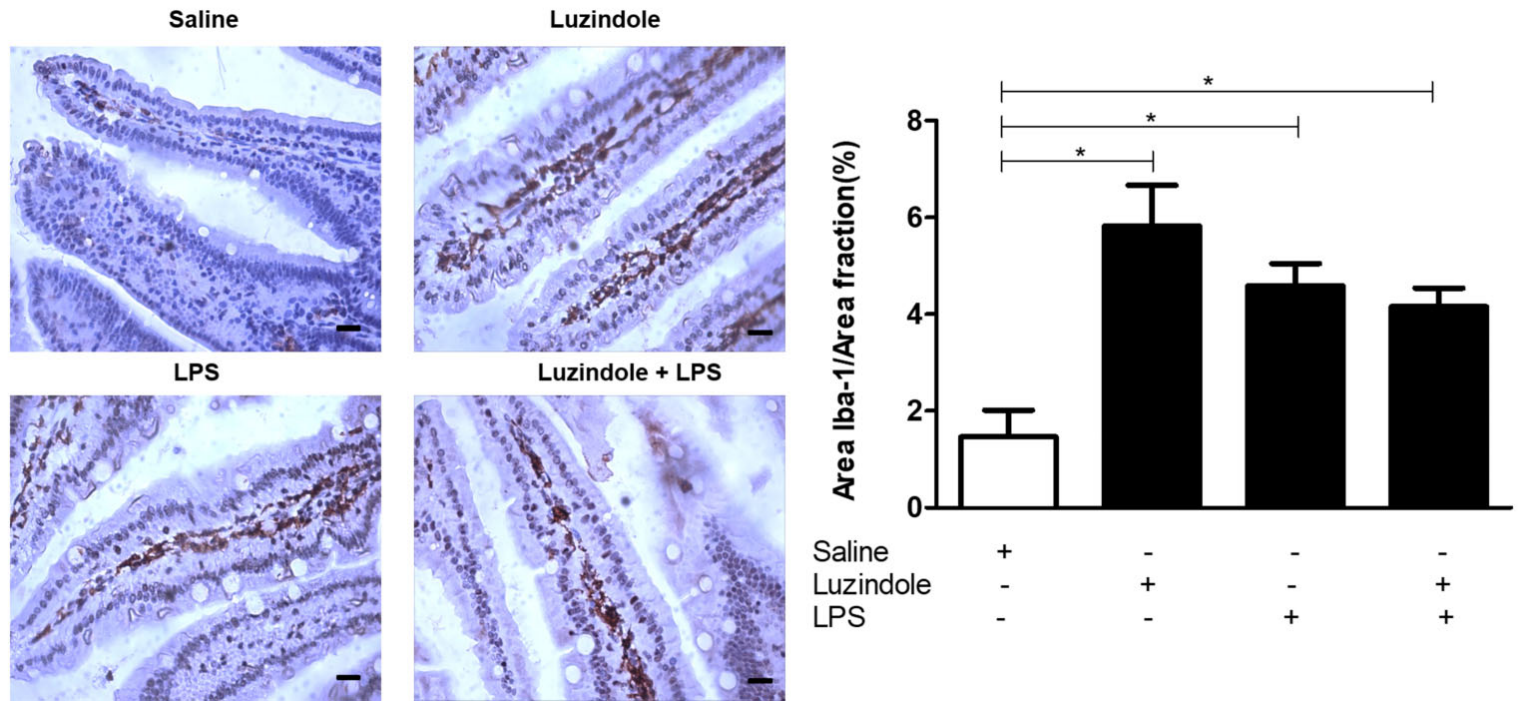
Representative histology of IBA-1 immunostaining in the experimental mouse jejunum. Iba-1-immunolabelled (400×) cells of Swiss mice receiving either saline (0.35 mg/kg, *ip*), Luzindole (0.35 mg/kg, *ip*), lipopolysaccharide (LPS) (1.25 mg/kg, *ip*), or Luzindole+LPS (0.35+1.25 mg/kg, *ip*). Scale bar=20 µm. Graph showing the stained area in relation to the total area of each group. Data are reported as means±SE. *P<0.05 (ANOVA followed by Bonferroni's test).

LPS, Luzindole, and Luzindole+LPS groups showed increased Iba-1 immunolabeling in the jejunum of mice compared to the saline group (P<0.05). There was no difference among the Luzindole, LPS, and Luzindole+LPS groups (P>0.05) (Figure 3).

#### IL-1β, TNF-α, and NF-kB immunolabeling, and MPO activity

**Figure 4:**
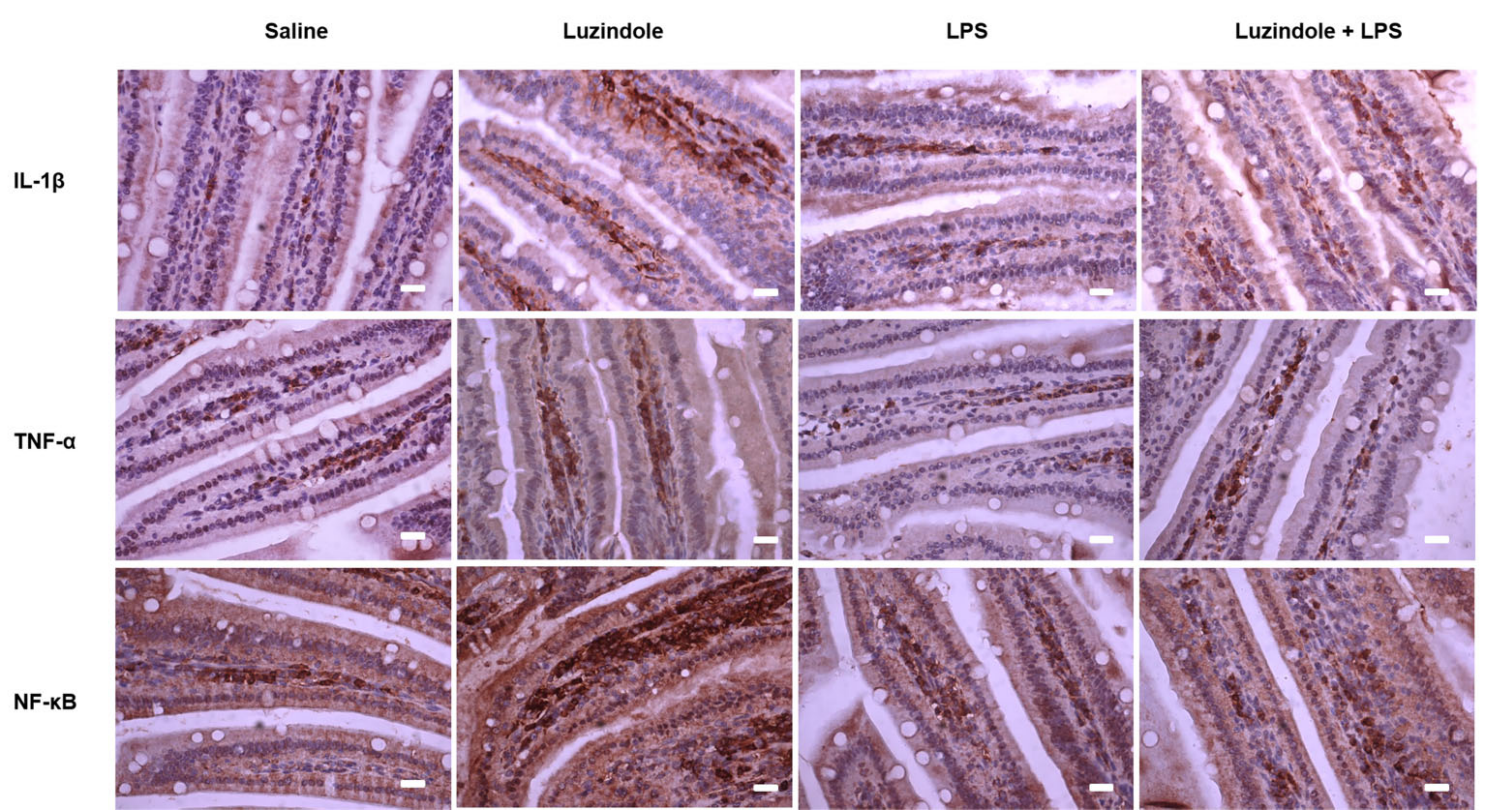
Jejunal interleukin (IL)-1β, tumor necrosis factor (TNF)-α, and nuclear factor (NF)-kB immunolabeling (400×) of Swiss mice receiving either saline (0.35 mg/kg, *ip*), Luzindole (0.35 mg/kg, *ip*), lipopolysaccharide (LPS) (1.25 mg/kg, *ip*), or Luzindole+LPS (0.35+1.25 mg/kg, *ip*). Scale bar=20 µm.

Greater IL-1β immunolabeling was observed in the Luzindole group compared to the saline group (P<0.05). However, no difference was found among the saline, LPS, and Luzindole+LPS groups (P>0.05) (Figure 4 and Table 3).

**Table 3:** Inflammatory markers of the experimental mice after treatment with saline, Luzindole, lipopolysaccharide (LPS), and Luzindole+LPS.

Markers	Saline	Luzindole	LPS	Luzindole+LPS
IL-1β (%)	2.01±0.36	4.90±0.61^a^	3.25±0.49	3.18±0.56
TNF-α (%)	1.43±0.23	3.88±0.52^a,b,c^	1±0.16	2.17±0.36
NF-kB (%)	0.43±0.07	2.01±0.27^a,b,c^	0.60±0.14	0.56±0.11
MPO (U/mg tissue)	0.24±0.09	0.07±0.00	0.86±0.21^a,d^	0.26±0.14

Data are reported as means±SE. The analyses of IL-1β, TNF-α, and NF-kB were made from the total area/immunostained fraction area. IL-1β: interleukin-1β; TNF-α: tumor necrosis factor-α; NF-kB: nuclear factor-kappa B; MPO: myeloperoxidase. ^a^P<0.05 compared with saline. ^b^P<0.05 compared with LPS. ^c^P<0.05 compared with Luzindole+LPS. ^d^P<0.05 compared with Luzindole (ANOVA followed by Bonferroni's test).

Jejunum from the Luzindole-treated group showed greater immunolabeling for TNF-α compared to the saline, LPS, and Luzindole+LPS groups (P<0.05). There was no difference among the saline, LPS, and Luzindole+LPS groups (P>0.05) (Figure 4 and Table 3).

NF-kB immunolabeling was higher in the Luzindole group compared to the saline, LPS, and Luzindole+LPS groups (P<0.05). No difference was found among the saline, LPS, and Luzindole+LPS groups (P>0.05) (Figure 4 and Table 3).

MPO enzymatic activity was higher in the LPS group compared to saline and Luzindole (P<0.05). There was no difference between the LPS and Luzindole+LPS groups, nor were there any differences among the saline, Luzindole, and Luzindole+LPS groups (P>0.05).

### Antioxidant activity

The effects of Luzindole (0.35 mg/kg, *ip*) on jejunum markers of oxidative stress is summarized in Table 4.

**Table 4:** Antioxidant activity of the experimental mice after treatment with saline, Luzindole, lipopolysaccharide (LPS), and Luzindole+LPS.

Markers	Saline	Luzindole	LPS	Luzindole+LPS
NP-SHs (μmol/mg tissue)	0.07±0.01	0.37±0.06^a^	0.18±0.008	0.21±0.02
CAT (mmol/min per mg tissue)	1.51±0.06	0.90±0.10^a^	1.32±0.05^b^	0.69±0.07^a^
MDA (nmol/mg tissue)	0.021±0.001	0.020±0.002	0.027±0.002	0.029±0.003
Nitrite/nitrate (µmol/mg protein)	0.22±0.003	0.15±0.001	0.42±0.07	0.23±0.06

Data are reported as means±SE. NP-SHs: non-protein sulfhydryls; CAT: catalase; MDA: malondialdehyde. ^a^P<0.05 compared with saline. ^b^P<0.05 compared with Luzindole+LPS (ANOVA followed by Bonferroni’s test).

There was an increase in NP-SHs levels in the Luzindole group compared to the saline group (P<0.05). There were no differences among the saline, LPS, and Luzindole+LPS groups, as well as among the Luzindole, LPS, and Luzindole+LPS groups (P>0.05).

Lower CAT activity was observed in the Luzindole and Luzindole+LPS groups compared to untreated controls (P<0.05). Lower CAT activity was found in the Luzindole+LPS group compared to the LPS group (P<0.05). There was no difference between the LPS and saline groups (P>0.05), Luzindole and LPS groups (P>0.05), as well as Luzindole and Luzindole + LPS groups (P>0.05)

No differences were found in MDA and nitrite/nitrate levels among all tested groups (P>0.05).

## Discussion

Endogenous MLT in the intestine may be essential for the prevention or treatment of diseases, such as colorectal cancer, ulcerative colitis, gastric ulcers, irritable bowel syndrome, and childhood enteric infections. To date, the blockade of endogenous MLT has not been extensively investigated. Preceding reports mainly analyzed the effects of Luzindole, a recognized MT1/MT2 receptor, in the presence of MLT supplementation (18). In this work, we evaluated the acute effects of Luzindole and LPS on the jejunum of mice. Such short-term effect has not been previously reported.

### Morphometric analysis

In the present study, acute treatment with Luzindole compromised the mucosa integrity early compared to unchallenged controls, confirming the negative impact of endogenous MLT blockade of MT1/MT2 receptors on intestinal homeostasis (10,19,20). Mice treated with LPS, a known endotoxin present in the outer membrane of Gram-negative bacteria, exhibited greater mucosa damage than those treated with Luzindole. Both LPS and Luzindole caused villus shortening compared to controls. Surprisingly, the association of LPS and Luzindole did not add to the intestinal mucosa injury. An explanation for this finding is that both Luzindole and MLT are acknowledged as pleiotropic regulators of inflammation (21). For instance, a work investigating the effects of Luzindole on LPS/d-galactosamine-induced acute hepatitis showed that Luzindole alleviated histological damage in the liver, reduced the level of transaminases in plasma, and improved survival.

Further analysis showed that Luzindole suppressed the production of the pro-inflammatory cytokines TNF-α and IL-6, inhibited the activation of caspase-3, -8, and -9, and suppressed the cleavage of caspase-3 and poly(ADP-ribose) polymerase (22). Our findings could be partially explained by the already documented anti-inflammatory property of Luzindole. Furthermore, the activation of the cytosolic MLT receptor MT3 (bypassing the Luzindole blockade of transmembrane MT1/MT2 receptors) should not be ruled out (10).

In consonance with intestinal villus blunting, marked necrotic crypt alterations were seen after LPS treatment. Unexpectedly, Luzindole-treated animals did not show an increase in necrotic crypt scores. Once more, the latter finding may be explained by the unopposed action of the MT3 receptors, already evidenced in another experimental setting (23). In the present work, mice treated with Luzindole, LPS, and Luzindole+LPS presented a reduction of goblet cell count, another marker of mucosa injury. Goblet cells are referred to as a healthy marker for intestinal epithelium, secreting components of the mucosal intestinal barrier. A reduction of this cell count negatively affects the mucous layer and exposes the epithelial surface to bacterial translocation (24,25).

### Inflammatory markers

In conformity with the impaired crypt architecture and villus shortening, MPO activity was increased in mice acutely treated with LPS. Our results partially agreed with those of Li et al. (26), who observed that mice challenged with LPS at the dose of 18 mg/kg and sacrificed 12 h after the injection showed MPO elevations compared to the saline group. Zielińska et al. (27), using a higher dose than the present study, showed that Luzindole (5 mg/kg, *ip*), administered 15 min before MLT, increased the activity of MPO in the gut of mice.

Our findings suggested that Luzindole treatment was not associated with marked leucocyte tissue infiltration, as seen by unaltered MPO activity (a marker of neutrophil azurophilic function), a result opposed to the LPS group. This lack of significant neutrophil tissue infiltration may be due to the acute effect of Luzindole on leukocyte diapedesis, either affecting leukocyte-endothelial transmigration or reducing blood vessel endothelial activation. In a zebrafish model, MLT has been found to enhance neutrophil blood-to-tissue migration, which was blocked by Luzindole (28). Noteworthy, most studies with MLT inhibitors examine histopathological effects within 24 h to 5 days after treatment (29). One previous report described significant intestinal inflammatory cell infiltration after 3 h (30).

In the present model, short-term exposure to LPS and Luzindole may have influenced inflammatory markers results. Previously, the differential effects of short-term versus long-term exposure to LPS, particularly regarding inflammation, have been demonstrated (31).

In our study, all groups treated with Luzindole, LPS, and Luzindole+LPS had higher immunostaining for Iba-1 than untreated controls. These findings suggested that Luzindole blockade of the MT1/MT2 receptors increased the activation of macrophages. In partial agreement with our findings, MLT supplementation, 3 h after LPS administration, reduced intestinal microvasculature and inflammation changes, probably due to a reduction in the local recruitment of immune cells (32). MLT anti‐inflammatory effects are expressed as both a decrease of the levels of inflammatory mediators, including IL‐6, IL‐8, COX‐2, and NO, and a reduction in paracellular permeability (33).

In association with Iba-1 immunostaining, inflammatory markers were also higher in the Luzindole group. The pronounced and consistent increase of the inflammatory cytokines TNF-α, IL-1β, and NF-kB support the Luzindole effect as a MLT blocker. MLT is associated with an inhibition of NF-kB signaling and a reduction of inflammatory cytokines expression (34).

MLT has been found to reduce inflammation in Caco-2 intestinal cells driven by IL-1β (33). In the study from Mannino et al. (33), 50 µM Luzindole did not prevent MLT (10-100 µM) inhibition of IL‐1β‐induced IL‐6 release in Caco-2 cells (33). Our findings suggested that Luzindole, per se, is a pro-inflammatory factor in the jejunum. This effect is likely related to the blockade of a baseline anti-inflammatory net function of MLT in the small intestine. It remains to be explored how Luzindole affects resident immune cells, such as Iba1-positive macrophages in the villus mucosa.

### Oxidative stress

The current work showed that in the Luzindole and Luzindole+LPS treated groups, the CAT antioxidant enzyme had significantly lower activity than in the saline group. MLT prevents the reduction of antioxidant enzymes. Therefore a lower activity of CAT after Luzindole is expected (35). CAT is important to control levels of hydrogen peroxide (H_2_O_2_). Luzindole has been shown to have a powerful antioxidant effect *in vitro* (36) and perhaps may have reduced H_2_O_2_ levels (even with LPS), and one may expect reduced CAT activity as well. Conversely, another possible explanation for the reduction of CAT activity in the Luzindole and Luzindole+LPS groups may be related to an oxidative environment within the mucosa and a higher usage of this enzyme without significant changes in cell lipid peroxidation, measured by MDA products. The former explanation seemed more plausible. Theoretically, unblocked MLT action through MT3 receptors reduced cell lipid peroxidation or direct and receptor-independent action of endogenous MLT had a protective cell effect. MLT has been shown to reduce MDA levels induced by LPS on duodenal segments (37).

MLT antioxidant action is also expressed as an increase of glutathione (GSH) content and activities of glutathione peroxidase (GPx), superoxide dismutase (SOD), and CAT (38). In this study, GSH was higher in the Luzindole than in the saline group. One plausible explanation for this finding is that endogenous MLT may use other mechanisms to bypass MT1/MT2 receptor signaling, e.g., via MT3 receptor, which has been found to protect against oxidative stress. Binding to quinone reductase and intracellular binding to calmodulin and tubulin and/or direct intracellular actions have all been advocated (5,6).

An *in vitro* study analyzed the effect of Luzindole on lipid peroxidation induced by LPS (400 mg/mL), with a higher dose than the one used in our study. Different concentrations of Luzindole (50-800 μm) were able to inhibit the formation of MDA, and this inhibitory effect was dose-dependent, i.e., the higher the dose, the greater the inhibition (19). On the other hand, a study with rats showed that the dose of indigo light (0.25 mg/kg, *ip*) at three different times (30 min, 6 h, and 12 h) administered after colitis induction reversed the protective effect of MLT, although without affecting MDA and GSH levels (39). However, Luzindole treatment did not increase lipid peroxidation activity in the jejunum, suggesting a direct protective effect or an MT3-dependent effect of MLT. Thus, MLT mucosal protection may have the participation of other mechanisms, i.e., quinone reductase or intracellular receptors. These findings are important, considering that a natural reduction of MLT is observed with aging (40).

Limitations must be acknowledged. This study evaluated only the very acute effects of Luzindole blockade of MT1/MT2 receptors (1.5 h after injection). Short-term administration of LPS, a recognized endotoxin, was used to draw a comparison. Previous evidence shows that short-term exposure *vs* long-term exposure produces different results. A comparison between acute effects and prolonged action would possibly provide additional information. To our knowledge, this was a unique study testing endogenous MLT suppression by the acute blockade of MT1/MT2 receptors in the small intestine in short-term exposure. Morphometric alterations and inflammatory and oxidative stress markers described herein may be explained both by the short-term exposure and by the bypassed action of other cytosolic and nuclear MLT receptors.

In conclusion, acute blocking of MT1/MT2 receptors by Luzindole resulted in precocious alterations of mucosa villus, although without significant leukocyte tissue infiltration (indirectly shown by reduction of jejunal MPO levels). We cannot rule out that a robust leucocyte infiltration may occur with a longer challenge. The lack of acute MPO activity by Luzindole may partly explain the better crypt necrotic scores seen with this group compared with the LPS-treated group alone. Furthermore, acute Luzindole treatment resulted in an early release of intestinal inflammatory markers. Luzindole may have a direct antioxidant activity as well. Our study reinforced the role of endogenous MLT in the protection of intestinal mucosa and corroborated the complex actions of Luzindole and MLT. The pleiotropic effects of MLT may be attributed to the unopposed actions of MLT on MT3 receptors. Endogenous MLT is essential for the constitutive preservation of the intestinal morphology, and awareness should be raised to the natural reduction of MLT associated with aging and its possible connection to increased risk for related intestinal disorders. More studies are warranted to determine the effects of Luzindole in neutrophil migration, bacterial translocation, endotoxemia, activation of mucosal immune cells, and the potential association with intestinal-related disorders.
